# Comparative Analysis of Antidepressant Mechanisms and Side Effects: Network Pharmacology and Molecular Docking Study of Amitriptyline, Mirtazapine, and Escitalopram for Treating Major Depressive Disorder

**DOI:** 10.1155/da/6981320

**Published:** 2026-10-02

**Authors:** Chuanjun Zhuo, Qiuyu Zhang, Haitao Song, Jiatong Zou, Ranli Li, Xiaoyan Ma, Ximing Chen, Yachen Li, Lei Yang, Lina Wang, Chao Li, Hongjun Tian

**Affiliations:** ^1^ Laboratory of Computational and Computational Psychiatry (CBCP_Lab) Tianjin Anding Hospital Mental Health Center of Tianjin Medical University Tianjin China; ^2^ Laboratory of Psychiatric-Neuroimaging-Genetic and Co-Morbidity (PGNC_Lab) Tianjin Anding Hospital Mental Health Center of Tianjin Medical University Tianjin China; ^3^ Department of Psychiatry Tianjin Fourth Center Hospital Tianjin China

**Keywords:** amitriptyline, escitalopram, major depressive disorder, mirtazapine, molecular docking, network pharmacology

## Abstract

**Background:**

Amitriptyline, mirtazapine, and escitalopram are effective antidepressants for treating major depressive disorder (MDD). Our study aimed to use computational biology to understand the mechanisms for the similarities and differences in efficacies and side effects of these drugs.

**Methods:**

Potential targets for MDD and the three drugs were obtained using public databases. Venn diagrams and protein–protein interaction (PPI) networks were constructed. Drug‐target‐pathway (D‐T‐P) networks were built using Cytoscape, and core targets were filtered using CytoNCA and CytoHubba plugins. Gene Ontology (GO) and Kyoto Encyclopedia of Genes and Genomes (KEGG) pathway analyses were conducted using the Database for Annotation, Visualization, and Integrated Discovery. Ligand compounds and receptor proteins were processed with PyMOL and AutoDock. The binding of amitriptyline, mirtazapine, and escitalopram with core targets was predicted by molecular docking using AutoDock Vina. The results were visualized using PyMOL and protein–ligand interaction profiler (PLIP).

**Results:**

Forty‐eight overlapping targets and 66, 50, and 30 individual targets for MDD treatment were identified for amitriptyline, mirtazapine, and escitalopram, respectively. Critical pathways included “Neuroactive ligand–receptor interaction,” “Calcium signaling pathway,” and “Serotonergic synapse.” Key targets, indicating a wide range of effects on cellular activities and signaling mechanisms, included D(2) dopamine receptor (DRD2), CYP2D6, monoamine oxidase type B (MAOB), solute carrier family 6 member 3 (SLC6A3), solute carrier family 6 member 4 (SLC6A4), 5‐hydroxytryptamine receptor 2C (HTR2C), and CHRNA4. MAOB had the highest binding affinity with amitriptyline, followed by SLC6A4 and DRD2. Binding stability was enhanced by salt bridges and π‐stacking interactions. Mirtazapine formed strong binding affinities with HTR2C, SLC6A4, and CHRNA4 through hydrogen bonds and π‐stacking interactions. Escitalopram exhibited the highest affinity for SLC6A3, followed by HTR2C.

**Conclusion:**

All three drugs regulated serotonin, norepinephrine (NE), and dopamine, but target specificity, pathways, and binding patterns differed. Amitriptyline exhibited stronger antidepressant effects and broader mechanisms but a higher risk of side effects. Mirtazapine’s dual mechanism regulates broad targets with reliable efficacy and fewer side effects. Escitalopram selectively acts on serotonin‐related targets, with narrow actions and potentially fewer side effects. These findings align with the clinical profiles of the antidepressant efficacy and side effects.

## 1. Introduction

Major depressive disorder (MDD) is one of the most common and severe mental illnesses globally. According to data from the Global Burden of Disease 2021 study, the age‐standardized prevalence rate of depressive disorder was 3440.1 per 100,000 people [1]. Between 2010 and 2021, anxiety disorders (16.7%) and depression (16.4%) were the leading causes of increased age‐standardized disability‐adjusted life year rates [2]. MDD causes significant psychological and physical distress to patients and imposes a heavy burden on healthcare systems and the economy [3]. Antidepressant medications are currently available for clinical treatment; however, patient responses and tolerance to these medications vary, resulting in less‐than‐ideal therapeutic outcomes.

In clinical practice, the efficacy of a drug is based on the degree of symptom reduction. Tricyclic antidepressants (TCAs), the earliest antidepressants, are still among the most effective drugs for treating MDD. However, TCAs are rarely used today due to their significant side effects. Selective serotonin reuptake inhibitors (SSRIs) are the first‐line antidepressants, although their efficacy is not outstanding. SSRIs may take several weeks to produce noticeable benefits, and their side effects, including dry mouth, nausea, constipation, and sexual dysfunction, are significant. A meta‐analysis in *The Lancet* analyzing the efficacy and acceptability of 21 antidepressants for MDD, which included 116,477 participants, demonstrated that amitriptyline, mirtazapine, and escitalopram are among the most effective antidepressants [4]. Based on this analysis, we aimed to explore the specific mechanisms and differences in the antidepressant effects of these three drugs using network pharmacology (NP) and molecular docking methods. Amitriptyline, a TCA, exhibits broad‐spectrum pharmacological effects and has been used to treat different types of depression. However, the use of amitriptyline has decreased due to the significant side effects [5–7]. Mirtazapine, a noradrenergic and specific serotonergic antidepressant, is well‐regarded for its efficacy and tolerance, particularly in patients with depression accompanied by anxiety symptoms [8, 9]. Escitalopram, a SSRI, is widely used for treating different types of depression due to its relatively few side effects and rapid onset of action [10, 11]. Despite the clinical effectiveness of these medications, individual variability in therapeutic outcomes, drug tolerance, and persistent side effects are a challenge [12].

Emerging research methods such as NP and molecular docking have been applied to study and develop antidepressant drugs [13–16]. NP involves constructing multilayer networks of diseases and drugs to reveal the multitarget and multipathway mechanisms of drug actions [17, 18]. Molecular docking uses computer simulations to model the interactions between drug molecules and target proteins, predict binding modes and affinities, and identify potential drug candidates. Molecular docking can shorten drug development timelines, reduce the number of experiments and resource consumption, and improve research reproducibility. Thus, NP and molecular docking are promising methods for exploring the pathogenesis and identifying new therapeutic targets for MDD [19]. This study employed NP and molecular docking techniques to explore the characteristics and differences in the effects of amitriptyline, mirtazapine, and escitalopram. The goal was to understand the reasons for differences in efficacy to develop personalized and prospective treatment strategies tailored to different preferences and individual variations. The research flowchart is shown in Figure 1.

**Figure 1:**
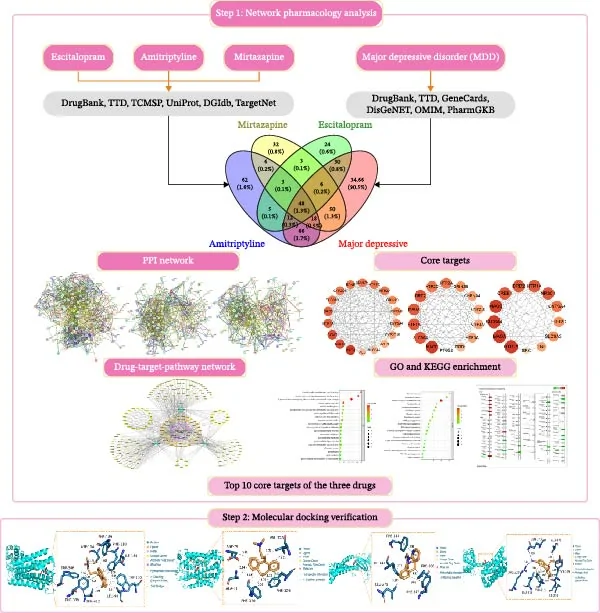
Network pharmacology flowchart. Step 1: Relevant targets of the three drugs and MDD were collected from multiple online public databases. Core targets were screened based on the PPI network diagram. GO and KEGG enrichment analyses were used to identify key biological processes and pathways. Step 2: The three drug molecules were docked with the 30 key targets (before filtering) obtained in the previous step for molecular docking validation.

Although previous studies have investigated the mechanisms of individual antidepressants using NP or molecular docking, few have conducted a systematic comparison of antidepressants representing different pharmacological classes within a unified analytical framework. To address this gap, our study uniquely integrates NP and molecular docking to compare amitriptyline, mirtazapine, and escitalopram at the target, pathway, and binding‐affinity levels. This approach allows us to identify both shared and drug‐specific mechanisms that may underlie their differences in clinical efficacy and tolerability. Accordingly, the main research questions of this study are as follows: (1) What are the common and distinct molecular targets and pathways associated with these three antidepressants? (2) How do their predicted binding affinities and interactions with key targets differ? (3) How might these mechanistic differences explain variations in therapeutic responses among patients?

Finally, at the end of this section, we briefly outline the structure of the article. Section 2 describes the data sources and analytical procedures used in the NP and molecular docking analyses. Section 3 presents the identified targets, enrichment analyses, and docking findings, followed by a detailed discussion of their implications. The article concludes with a summary of the main findings and future research directions.

## 2. Methods

### 2.1. Screening of Related Targets for MDD and Drugs

The drug targets for amitriptyline, mirtazapine, and escitalopram were identified using the DrugBank (https://go.drugbank.com/) [20], Therapeutic Target (TTD: https://db.idrblab.net/ttd/) [21], Traditional Chinese Medicine Systems Pharmacology (TCMSP: https://tcmsp-e.com/) [22], UniProt platform (https://www.uniprot.org/) [23], Drug–Gene Interaction (DGIdb: https://www.dgidb.org/) [24], and TargetNet (http://targetnet.scbdd.com/) databases [25]. The Simplified Molecular Input Line Entry System (SMILES) numbers for these drugs were obtained from PubChem (https://pubchem.ncbi.nlm.nih.gov/) [26] and entered into the SwissTargetPrediction database [27] (http://swisstargetprediction.ch/) to predict related protein targets. The relevant targets of the three drugs were retrieved from these databases, and duplicates were removed to obtain three distinct drug target datasets.

To identify disease‐related targets in *Homo sapiens*, the keyword “Major Depressive Disorder” was searched in DrugBank, TTD, GeneCards (https://www.genecards.org/) [28], DisGeNET (https://www.disgenet.org/) [29], Online Mendelian Inheritance in Man (OMIM: https://omim.org/) [30], and Pharmacogenomics Knowledgebase (PharmGKB: https://www.pharmgkb.org/) [31]. All potential targets were combined, and duplicates were removed to obtain the “Major Depressive Disorder” disease‐related target dataset. A Venn diagram was created using the Venny_v2.1.0 platform (https://bioinfogp.cnb.csic.es/tools/venny/index.html) to visualize the connections between the disease targets for amitriptyline, mirtazapine, and escitalopram.

### 2.2. Construction of the Protein–Protein Interaction (PPI) Networks and the Drug‐Target‐Pathway (D‐T‐P) Network

Shared targets were designated as the therapeutic targets for drugs to treat MDD, and PPI networks were built using the STRING_v12.0 database (https://string-db.org/) [32]. The data were exported in the TSV format and imported into Cytoscape_v3.9.0 software [33] for visualization and analysis. Key topological parameters, including the degree of centrality [34], closeness centrality [35], and betweenness [36], were analyzed through the CytoNCA [37] plug‐in within Cytoscape to prioritize the targets. The prioritized targets were used to construct an undirected network. Core genes were identified based on the maximum cluster centrality and maximum neighborhood composition metrics using the CytoHubba [36] plug‐in. The top 10 core targets for the treatment of MDD with amitriptyline, mirtazapine, and escitalopram were selected based on the PPI networks, the D‐T‐P network, and core target screening results.

### 2.3. Enrichment Analysis of Target Genes Involved in MDD Treatment With Amitriptyline, Mirtazapine, and Escitalopram

Gene Ontology (GO) enrichment analyses were conducted using the Database for Annotation, Visualization, and Integrated Discovery [38, 39] (DAVID_v6.8: https://david.ncifcrf.gov/), which offers detailed and systematic annotation of the biological functions of large‐scale genes or proteins. The biological processes (BPs), cellular components (CCs), and molecular functions (MFs) with significant biological annotations (*p*‐value < 0.05) and a false discovery rate (FDR) <0.1 were selected for further analysis. Pathway enrichment analysis was performed using the Kyoto Encyclopedia of Genes and Genomes (KEGG) [40] (http://www.kegg.jp/) database. The enrichment analysis results were visualized using an online bioinformatics analysis platform (http://www.bioinformatics.com.cn/).

### 2.4. Molecular Docking Verification

Molecular docking was performed to predict the binding affinities between the three antidepressants (amitriptyline, mirtazapine, and escitalopram) and the core target proteins using AutoDock Tools 1.5.6 (https://autodock.scripps.edu/) and AutoDock Vina (https://vina.scripps.edu/) [41].

### 2.5. Ligand Preparation

The three‐dimensional structures of amitriptyline, mirtazapine, and escitalopram were obtained from the PubChem database in the SDF format. Each ligand was converted to MOL2 format, imported into ChemBio3D Ultra 14.0 for energy minimization using the MM2 force field, and then saved as a Protein Data Bank (PDB) file. Finally, the ligands were processed with AutoDock Tools 1.5.6 and saved in PDBQT file.

### 2.6. Protein Preparation

The crystal structures of the core target proteins were obtained from the RCSB PDB (https://www.rcsb.org/) [42]. Using PyMOL v2.4.1 (https://pymol.org/2/), all water molecules and cocrystallized ligands were removed. The protein structures were then imported into AutoDock Tools 1.5.6, where hydrogen atoms were added, charges were calculated, and atom types were assigned. The resulting structures were saved in the PDBQT format.

### 2.7. Grid Preparation

Binding pockets for each target protein were predicted using POCASA v1.1 (https://g6altair.sci.hokudai.ac.jp/g6/service/pocasa/). Using AutoDock Tools, the grid box center coordinates and box dimensions were manually adjusted to ensure that the grid box fully enclosed the predicted pocket.

### 2.8. Molecular Docking

Molecular docking was performed using AutoDock Vina. The exhaustiveness parameter was set to 100, and the num_modes parameter was explicitly set to 9, generating nine binding conformations per ligand–protein pair. The conformation with the highest binding affinity (i.e., the most negative binding energy score, kcal/mol) was selected for subsequent analyses. The results were visualized using PyMOL_v2.4.1 and the online protein–ligand interaction profiler (PLIP) platform [43].

## 3. Results

### 3.1. Drug Targets for Treating MDD and the Venn Diagrams

A comprehensive search of the DrugBank, TTD, TCMSP, UniProt, DGIdb, TargetNet, and SwissTargetPrediction databases was performed, and duplicates were removed, resulting in 220 targets for amitriptyline, 166 targets for mirtazapine, and 131 targets for escitalopram. After merging the screening results for the six databases and removing duplicates, 3696 disease‐related targets for MDD were identified. The targets of amitriptyline, mirtazapine, and escitalopram were compared with the MDD gene set, and overlapping targets were identified. Target information was validated via the UniProt database (Figure 2A), yielding 66 targets for amitriptyline, 50 targets for mirtazapine, and 30 targets for escitalopram. Eighteen common targets for amitriptyline and mirtazapine, 12 for amitriptyline and escitalopram, six for mirtazapine and escitalopram, and 48 for all three drugs for treating MDD were identified, as shown in Table 1.

Figure 2Potential targets of amitriptyline, mirtazapine, and escitalopram in the treatment of MDD. (A) The Venn diagram of the intersection of MDD targets and the targets of the three drugs shows 48 overlapping targets, with 18 targets shared between amitriptyline and mirtazapine, six targets shared between mirtazapine and escitalopram, and 12 targets shared between amitriptyline and escitalopram. PPI networks for amitriptyline (B), mirtazapine (C), and escitalopram (D). CytoNCA was used to identify the core drug targets for MDD treatment for amitriptyline (E), mirtazapine (F), and escitalopram (G). CytoHubba was used to screen core drug targets, specifically MCC for amitriptyline (H), MHC for amitriptyline (I), MCC for mirtazapine (J), MHC for mirtazapine (K), MCC for escitalopram (L), and MHC for escitalopram (M).

**Figure:**
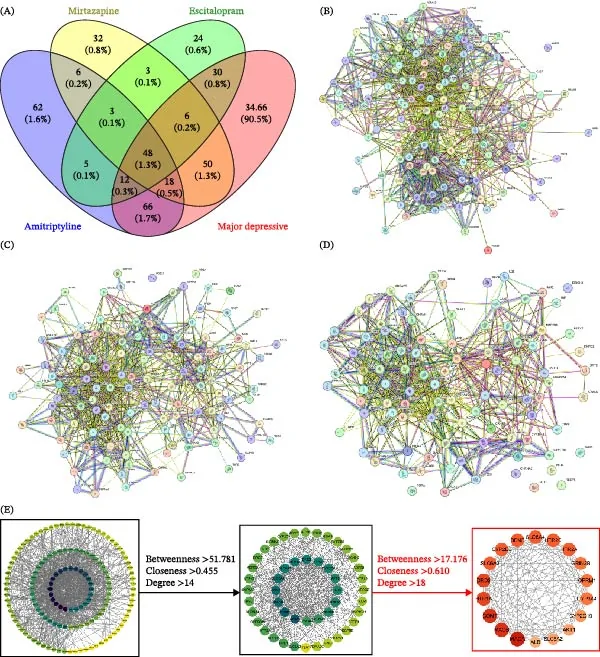

**Figure:**
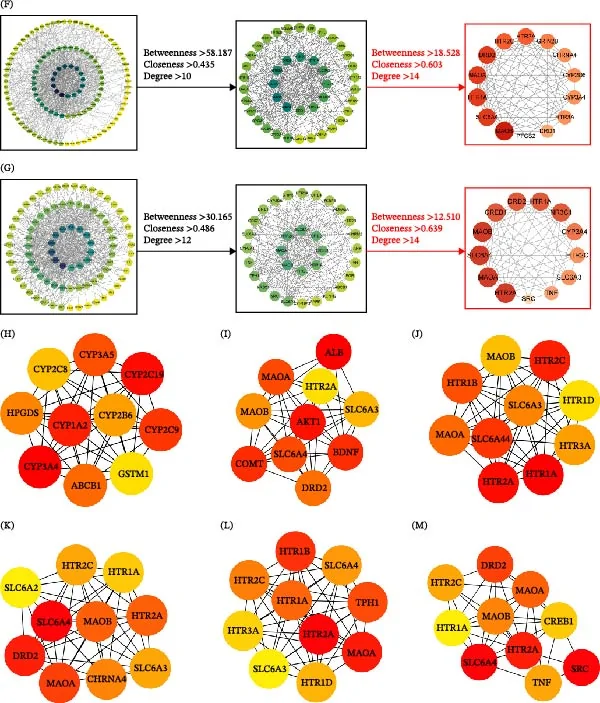

**Table 1:** Related targets for drugs treating MDD.

Drugs	Targets
Common targets of the three drugs	ADRA1A, ADRA1B, ADRA1D, ADRA2A, ADRA2B, ADRA2C, CASP9, CHRM1, CHRM2, CHRM4, CYP19A1, CYP2C19, CYP2C9, CYP2D6, CYP3A4, DRD1, DRD2, DRD3, DRD4, DRD5, GALR3, HRH1, HRH2, HRH3, HRH4, HSD11B1, HTR1A, HTR1B, HTR1D, HTR1E, HTR1F, HTR2A, HTR2B, HTR2C, HTR3A, HTR5A, KCNH2, MAOA, MAOB, MAPK14, NOS3, OPRL1, RORA, SIGMAR1, SLC6A2, SLC6A3, SLC6A4, TAAR1
Amitriptyline and mirtazapine	ADRB1, ADRB2, AHR, CACNA1B, CHRM3, CHRM5, CHRNA4, CYP1A2, GRIN2B, HTR6, HTR7, JAK3, NR2F2, OPRK1, OPRM1, PLA2G1B, PRL, SMO
Amitriptyline and escitalopram	ABCB1, APP, CYP17A1, DPP4, GRIK2, GRIK4, KIF11, MIF, P2RX7, PGR, SCN5A, SMPD1
Mirtazapine and escitalopram	GSK3B, JAK2, NOS1, NPY5R, PRCP, SIRT2
Amitriptyline	AANAT, AARS1, ABCG2, ACHE, ADA, ADRB3, AKT1, ALB, ALOX5, ALPL, AMD1, AMPD3, BCHE, BCL2A1, BDNF, CACNG2, CASR, CDK4, COMT, CRHR1, CYBB, CYP2B6, CYP2C8, CYP3A5, DDIT4, DNMT1, FGFR1, GNAS, GSTA1, GSTM1, GSTP1, HDAC4, HPGDS, HSD17B3, IFNG, KCNA1, KCNK2, KCNQ2, KCNQ3, KDM1A, KDR, NMDAR, NR1H3, NTRK1, NTRK2, OPRD1, ORM1, PDGFRA, PDGFRB, PRKCB, PRKCE, PRKCG, PRNP, RCOR1, RELA, RET, RGS4, SCN2A, SCN9A, SLC18A2, TBXA2R, TERT, TRPV1, UGT1A4, UGT2B10, UGT2B7
Mirtazapine	ABL1, ACE, ADORA2A, CACNA1H, CCR5, CHRFAM7A, CHRNA3, CHRNA7, CPT2, CSNK1D, CSNK1E, CTSK, CYP11B1, CYP11B2, DYRK1A, EPHX2, GABRA1, GABRA2, GABRA5, GABRB3, GAL, GRM2, GRM4, HCRTR1, HCRTR2, HTR4, LEP, MAPK9, MET, MME, MMP1, MMP3, MMP9, MPO, MYLK, NOS2, PDE2A, PDE4B, PIK3CD, PIK3CG, PPARG, PREP, PSEN2, PTGS2, SCD, SLC22A2, TGFBR1, THRB, TPH2, TSPO
Escitalopram	AOX1, AR, BMP5, CACNA1C, CDK9, CHRNA1, CREB1, CRHR2, CSNK2A1, DTNBP1, ERICH3, FKBP5, GLDC, GRIA3, GRK5, GRM5, IL11, JAK1, KDM4A, MAP2K1, NR3C1, NR3C2, PARP1, REEP5, SERPINE1, SRC, TACR1, TNF, TPH1, XIAP

The potential targets for treating MDD with the three drugs were imported into the STRING database to generate three PPI network diagrams (Figure 2B,D). The downloaded TSV files were input into Cytoscape for PPI network reconstruction. The network for amitriptyline consisted of 141 connected key nodes and 1218 edges. The core targets were screened using the CytoNCA plugin based on betweenness, closeness, and degree centrality greater than the median (51·781, 0·455, and 14). Targets with all three values greater than the median (17·176, 0·610, and 18) were further screened, resulting in 18 potential core MDD targets for amitriptyline (Figure 2E). The network for mirtazapine consisted of 119 nodes and 820 edges, and the two‐step screening process yielded 14 potential core targets for treating MDD (betweenness, closeness, and degree values: 58.187, 0.435, and 10 and 18.528, 0.603, and 14, respectively, for the two steps) (Figure 2F). The network for escitalopram consisted of 89 nodes and 637 edges, and 13 potential core targets for escitalopram were obtained through the two‐step screening process (betweenness, closeness, and degree values: 30.165, 0.486, and 12; and 12.510, 0.639, and 14, respectively, for the two steps) (Figure 2G). The CytoHubba plugin was used to screen the core targets for the three drugs, and maximum cluster centrality and maximum neighborhood composition metrics were used to identify the six core target clusters for amitriptyline (Figure 2H,I), mirtazapine (Figure 2J,K), and escitalopram (Figure 2L,M).

### 3.2. MDD Drug Target Enrichment Analysis and the D‐T‐P Compound Network Diagram

GO enrichment analysis indicated that “chemical synaptic transmission” and “G protein‐coupled receptor signaling pathway” are the most significant BPs affected by amitriptyline, mirtazapine, and escitalopram (Figure 3A,E,I). “Plasma membrane,” “dendrite,” and “synapse” are the most critical CCs (Figure 3B,F,J), and “cytoplasm” also plays a key role for escitalopram. “G protein‐coupled serotonin receptor activity,” “serotonin binding,” and “neurotransmitter receptor activity” are the most prominent MFs (Figure 3C,G,K). “Neuroactive ligand–receptor interaction” (Figure 4B) is the most important pathway for all three drugs (Figure 3D,H,L and Table 2). “Calcium signaling pathway” (Figure 4C) and “Serotonergic synapse” are almost equally important for amitriptyline and mirtazapine, and “Serotonergic synapse” is crucial for the effects of escitalopram (Figure 4A).

Figure 3GO and KEGG analyses. (A–D) GO biological process (BP), cellular component (CC), molecular function (MF), and KEGG pathway analysis of amitriptyline’s antidepressant action. (E–H) BP, CC, MF, and KEGG analyses of mirtazapine’s antidepressant action. (I–L) BP, CC, MF, and KEGG analyses of escitalopram’s antidepressant action. The larger the bubble, the more genes are enriched, and the redder the color, the more significant the enrichment. (M) “Drug‐Target‐Pathway” network diagram. Red represents the disease, green represents the drugs, yellow and orange represent the potential targets, and pink represents the pathways. The three yellow sectors show the independent targets of the three drugs, and the three yellow hollow circles represent shared targets between two adjacent drugs. The orange double circle in the center represents the common potential targets of all three drugs for treating MDD.

**Figure:**
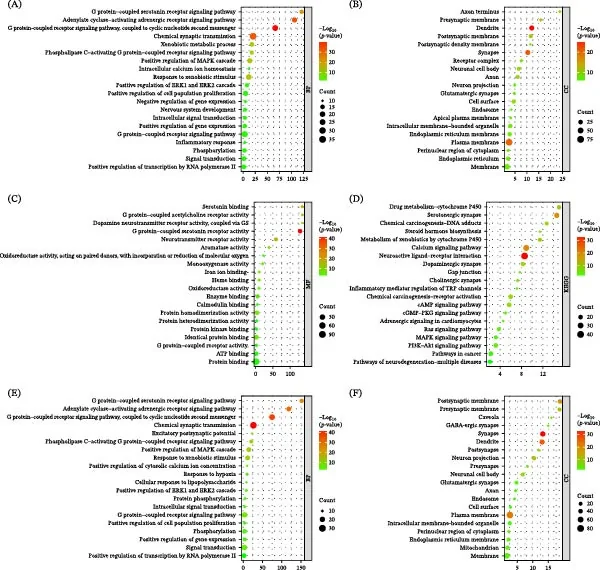

**Figure:**
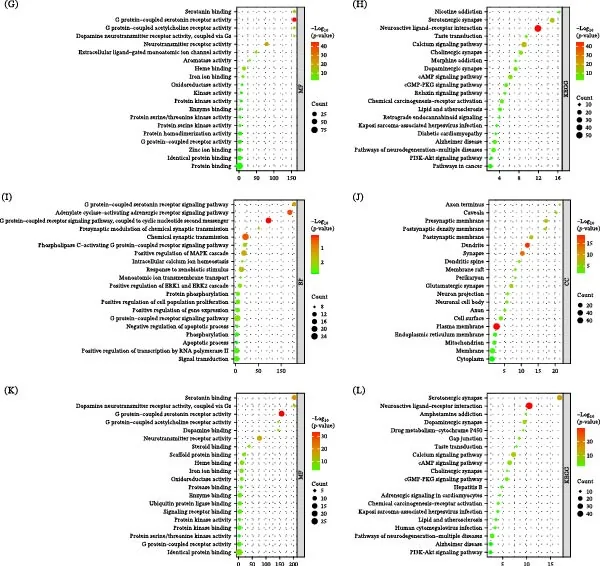

**Figure:**
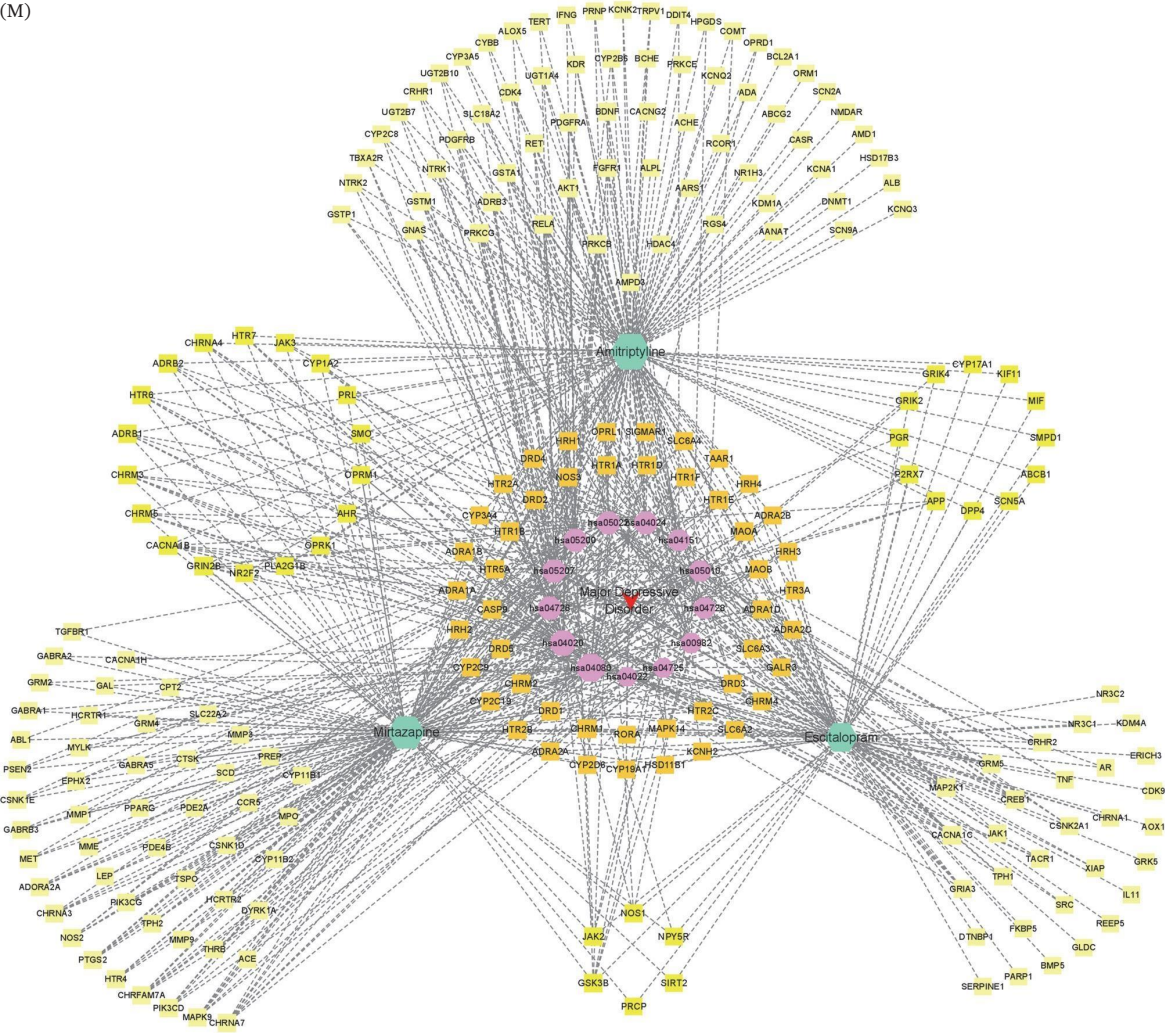

Figure 4(A) “Neuroactive ligand–receptor interaction” pathway diagram. (B) “Calcium signaling pathway” pathway diagram. Red represents the targets influenced by all three drugs, pink represents the targets influenced by two drugs, and green represents the targets influenced by one drug. (C) “Serotonergic synapse” pathway diagram. Red represents the targets influenced by escitalopram.

**Figure:**
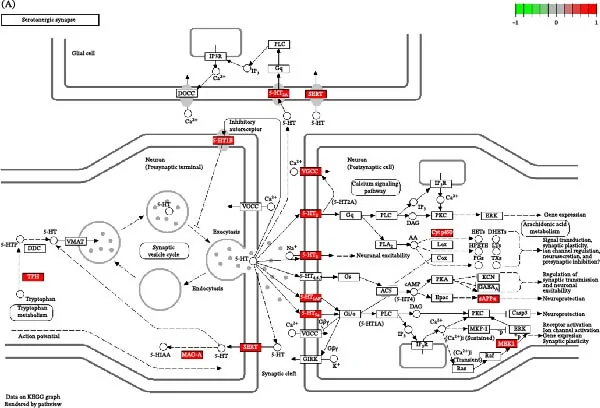

**Figure:**
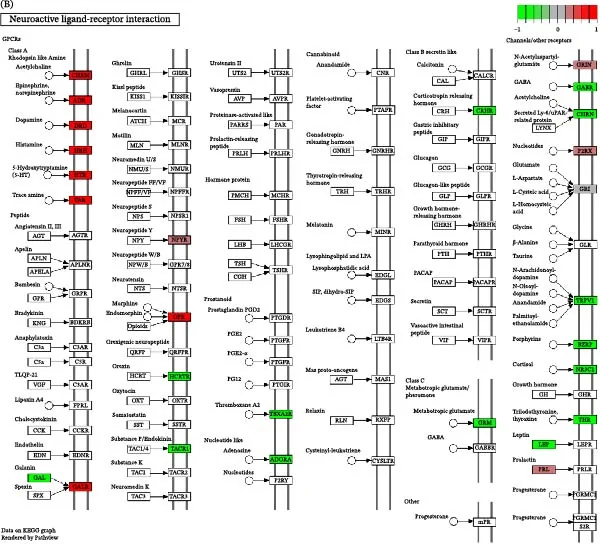

**Figure:**
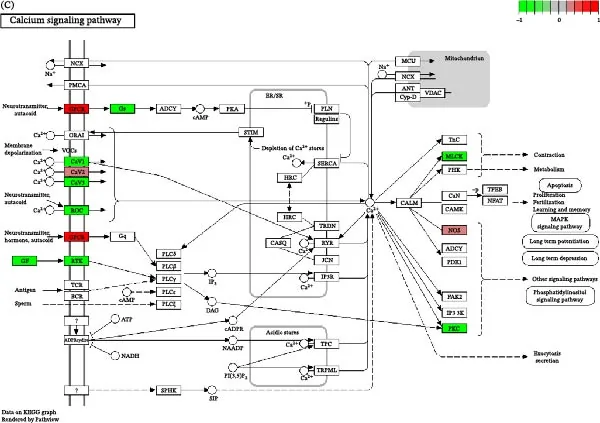

**Table 2:** KEGG enrichment results of key genes.

Drug	ID	Term	Count	Gene
Common pathways of three drugs	hsa04080	Neuroactive ligand–receptor interaction	73	TAAR1, ADRA2A, OPRL1, HRH1, GALR3, HRH3, HRH2, HRH4, DRD1, DRD2, DRD3, DRD4, DRD5, HTR1E, HTR1F, HTR1D, HTR1A, HTR1B, HTR5A, CHRM3, CHRM5, PRL, HTR6, HTR7, OPRM1, CHRNA4, ADRB1, ADRB2, OPRK1, GRIN2B, GRIK4, GRIK2, P2RX7, NPY5R, ADRB3, OPRD1, CRHR1, TBXA2R, TRPV1, GABRB3, THRB, HTR4, GRM2, GRM4, TSPO, GAL, ADORA2A, CHRNA3, CHRNA7, GABRA2, GABRA1, GABRA5, HCRTR2, HCRTR1, LEP, CHRFAM7A, CHRNA1, NR3C1, CRHR2, GRM5, GRIA3, TACR1
	hsa04020	Calcium signaling pathway	47	CHRM2, CHRM1, HTR2B, HTR2C, ADRA1D, HTR2A, ADRA1B, ADRA1A, HRH1, HRH2, DRD1, DRD5, NOS3, HTR5A, CHRM3, CHRM5, CACNA1B, ADRB1, ADRB2, HTR6, HTR7, GRIN2B, P2RX7, NOS1, RET, TBXA2R, KDR, PRKCG, PDGFRB, NTRK1, PDGFRA, NTRK2, PRKCB, ADRB3, GNAS, FGFR1, CHRNA7, HTR4, CACNA1H, MYLK, NOS2, ADORA2A, CHRFAM7A, MET, CACNA1C, TACR1, GRM5
	hsa04726	Serotonergic synapse	33	MAOB, MAOA, HTR2B, HTR2C, HTR2A, CYP2C19, SLC6A4, CYP2D6, HTR1E, HTR1F, HTR1D, HTR1A, HTR1B, HTR3A, HTR5A, CYP2C9, CACNA1B, HTR6, HTR7, APP, ALOX5, SLC18A2, PRKCG, PRKCB, CYP2C8, GNAS, GABRB3, TPH2, HTR4, PTGS2, MAP2K1, TPH1, CACNA1C
	hsa05207	Chemical carcinogenesis–receptor activation	32	CYP3A4, CHRNA4, CACNA1B, ADRB1, AHR, ADRB2, CYP1A2, PGR, JAK2, UGT2B10, PRKCG, GSTM1, PRKCB, RELA, CYP2B6, ADRB3, GSTA1, GNAS, UGT1A4, AKT1, UGT2B7, CHRNA3, EPHX2, CHRNA7, PIK3CD, CHRFAM7A, AR, MAP2K1, CREB1, SRC, XIAP, CACNA1C
	hsa04024	cAMP signaling pathway	30	CHRM2, CHRM1, HTR1E, HTR1F, HTR1D, HTR1A, HTR1B, DRD1, DRD2, DRD5, ADRB1, ADRB2, GRIN2B, HTR6, BDNF, RELA, CRHR1, GNAS, AKT1, KCNK2, PIK3CD, HTR4, MAPK9, ADORA2A, PDE4B, MAP2K1, CACNA1C, CRHR2, CREB1, GRIA3
	hsa05022	Pathways of neurodegeneration–multiple diseases	30	CHRM1, SIGMAR1, MAPK14, SLC6A3, CASP9, CHRM3, CHRM5, CACNA1B, GRIN2B, APP, GSK3B, PRKCG, PRNP, PRKCB, BDNF, CYBB, RELA, NOS2, CHRNA7, PSEN2, CSNK1E, PTGS2, MAPK9, MAP2K1, CSNK2A1, CACNA1C, TNF, GRM5, NOS1, GRIA3
	hsa04728	Dopaminergic synapse	22	MAOB, MAOA, MAPK14, SLC6A3, DRD1, DRD2, DRD3, DRD4, DRD5, CACNA1B, GRIN2B, GSK3B, PRKCG, PRKCB, COMT, GNAS, AKT1, SLC18A2, MAPK9, CACNA1C, CREB1, GRIA3
Amitriptyline and mirtazapine	hsa05200	Pathways in cancer	31	CASP9, SMO, JAK3, PDGFRB, PRKCG, NTRK1, RET, PDGFRA, GSTM1, PRKCB, GSTP1, RELA, IFNG, TERT, CDK4, GSTA1, GNAS, AKT1, FGFR1, GSK3B, NOS2, MMP1, PIK3CD, PTGS2, MMP9, TGFBR1, MAPK9, ABL1, PPARG, JAK2, MET
Amitriptyline and escitalopram	hsa04151	PI3K‐Akt signaling pathway	23	CHRM2, CHRM1, NOS3, CASP9, PDGFRB, NTRK1, RET, PDGFRA, NTRK2, BDNF, PRL, RELA, CDK4, DDIT4, KDR, AKT1, JAK3, FGFR1, GSK3B, MAP2K1, CREB1, JAK2, JAK1
Mirtazapine and escitalopram	hsa05010	Alzheimer disease	22	GSK3B, CHRM1, CASP9, NOS1, CHRM3, NOS2, MME, CHRNA7, CHRM5, PSEN2, PIK3CD, CSNK1E, PTGS2, GRIN2B, MAPK9, CHRFAM7A, APP, GRM5, MAP2K1, CSNK2A1, CACNA1C, TNF
Amitriptyline	hsa00982	Drug metabolism–cytochrome P450	17	UGT2B10, GSTM1, MAOB, MAOA, GSTP1, CYP3A4, CYP2C19, CYP3A5, CYP2C9, HPGDS, CYP2C8, CYP2B6, CYP2D6, CYP1A2, GSTA1, UGT1A4, UGT2B7
Mirtazapine	hsa04725	Cholinergic synapse	13	CHRM2, CHRM3, CHRNA3, CHRM1, CHRNA4, CHRM4, CHRNA7, CHRM5, CACNA1B, PIK3CD, PIK3CG, CHRFAM7A, JAK2
Escitalopram	hsa04022	cGMP‐PKG signaling pathway	10	MAP2K1, CREB1, NOS3, ADRA1D, CACNA1C, ADRA1B, ADRA2C, ADRA1A, ADRA2B, ADRA2A

The basic mechanism of action for most antidepressants is based on the theory of neurotransmitter synaptic transmission. Escitalopram is a typical serotonin (5‐HT) reuptake inhibitor. The activation of the 5‐HT1A receptor may be involved in the action of many antidepressants, including SSRIs. Amitriptyline blocks 5‐HT and norepinephrine (NE) reuptake, thereby increasing the concentration of these neurotransmitters in the synaptic cleft. Amitriptyline also antagonizes other neurotransmitter receptors, including the 5‐HT2, NE α1, and histamine H1 receptors. Mirtazapine primarily blocks α2 receptors, increasing the release of NE and 5‐HT. All of these receptors belong to the G protein‐coupled receptor (GPCR) family, and the BPs involved in the actions of antidepressants are closely related to ligand‐receptor interactions within the nervous system.

A D‐T‐P network diagram (Figure 3M) that includes the disease, three drugs, 48 common targets, 36 common targets of two drugs, 146 interconnected independent targets, and 13 significant potential pathways was constructed. The network consists of 247 nodes and 747 edges.

### 3.3. Molecular Docking Verification

The top 10 target proteins were selected based on their degree values for molecular docking with amitriptyline, mirtazapine, and escitalopram. Tables 3–5 show the best binding affinities between receptors and ligands, ranging from −5.7 to −9.9 kcal/mol. The average binding energy for the top 10 target proteins with amitriptyline was −7.52 kcal/mol (range −5.7 to −9.9 kcal/mol). Monoamine oxidase type B (MAOB) had the strongest affinity for amitriptyline, with a binding energy of −9.9 kcal/mol. Sodium‐dependent serotonin transporter (solute carrier family 6 member 4 [SLC6A4]) and D(2) dopamine receptor (DRD2) also exhibited strong binding affinities (binding energies of −8.8 and −8.2 kcal/mol, respectively). These results indicate that MAOB is the most important target for amitriptyline, followed by SLC6A4 and DRD2. These molecules may play key roles in the antidepressant effects of amitriptyline. As shown in Table 4, the average binding energies of the 10 target proteins with mirtazapine were −7.68 kcal/mol (range −7.0 to −8.6 kcal/mol). Mirtazapine exhibited the highest binding affinity for 5‐hydroxytryptamine receptor 2C (HTR2C) (−8.6 kcal/mol). As shown in Table 5, the average binding energy of the 10 target proteins with escitalopram was −7.17 kcal/mol (range −6.1 to −8.1 kcal/mol). Sodium‐dependent dopamine transporter (solute carrier family 6 member 3 [SLC6A3]) had the strongest affinity for escitalopram (−8.1 kcal/mol).

**Table 3:** The docking results of the 10 core proteins with amitriptyline.

Number	Gene symbol	PDB ID	Degree	Affinity
1	MAOA	2z5y	34	−7·1
2	COMT	6i3c	33	−6·0
3	BDNF	1bnd	31	−5·7
4	MAOB	1s3e	31	−9·9
5	SLC6A4	5i6x	29	−8·8
6	SLC6A3	8y2e	28	−7·8
7	DRD2	6cm4	28	−8·2
8	HTR1A	8fyt	28	−6·4
9	CYP2D6	6csd	27	−7·7
10	HTR2A	7wc8	27	−7·6

**Table 4:** The docking results of the 10 core proteins with mirtazapine.

Number	Gene symbol	PDB ID	Degree	Affinity
1	MAOB	1s3e	29	−7·5
2	MAOA	2z5y	29	−7·1
3	SLC6A4	5i6x	28	−7·9
4	HTR2C	6bqh	26	−8·6
5	HTR2A	7wc8	26	−7·6
6	DRD2	6cm4	26	−8·0
7	HTR1A	8fyt	25	−7·0
8	CYP2D6	6csd	21	−7·8
9	CHRNA4	5kxi	20	−7·8
10	GRIN2B	7ujr	20	−7·5

**Table 5:** The docking results of the 10 core proteins with escitalopram.

Number	Gene symbol	PDB ID	Degree	Affinity
1	MAOB	1s3e	23	−6·9
2	MAOA	2z5y	22	−7·2
3	SLC6A4	5i6x	21	−7·2
4	HTR2A	7wc8	21	−7·0
5	DRD2	6cm4	20	−7·4
6	HTR2C	6bqh	19	−7·4
7	HTR1A	8fyt	19	−6·1
8	SLC6A3	8y2e	19	−8·1
9	NR3C1	4udd	18	−7·6
10	SRC	1y57	18	−6·8

The molecular docking results indicate that MAOB and SLC6A4 are the most significant targets for amitriptyline treatment of MDD. The affinities of amitriptyline for these targets suggest that the strong antidepressant effects of amitriptyline are related to 5‐HT and monoamine oxidase transport and function. The core targets exhibit strong binding affinities with mirtazapine, suggesting that mirtazapine has reliable antidepressant effects and may be effective in a wider range of patients; however, the side effects should be monitored. The molecular docking results for escitalopram demonstrate that the binding of the drug to the core targets is not as strong as amitriptyline and mirtazapine binding to the core targets. However, escitalopram still shows good affinities for DA and 5‐HT signaling. Although the molecular docking results align with clinical experience, the high affinity of mirtazapine for many targets was unexpected. Given the higher incidence of side effects associated with amitriptyline, mirtazapine should be considered more in clinical practice, provided that contraindications are ruled out and side effects are monitored.

Figure 5A–N shows the binding energy results. As shown in Table 6, amitriptyline binds to SLC6A4 and DRD2 through salt bridge, π‐stacking (perpendicular), and hydrophobic interactions. Thus, amitriptyline forms stable ionic bonds and perpendicular aromatic ring interactions with amino acid residues of the targets and forms hydrophobic contacts, which enhance the binding stability between the drug and the target. Amitriptyline also interacts with MAOB and SLC6A3 through π‐stacking (parallel) and hydrophobic interactions, suggesting that amitriptyline’s aromatic ring stacks in a parallel manner with the aromatic residues of the protein targets. Combined with the hydrophobic interactions, the π‐stacking increases the binding affinity of amitriptyline. These findings support the strong antidepressant effects and multiple mechanisms of action of amitriptyline, including the regulation of SLC6A4 (related to 5‐HT transport), DRD2 (dopamine receptor), MAOB (monoamine oxidase B), and SLC6A3 (dopamine transporter), which are likely key targets for the antidepressant effects of amitriptyline.

Figure 5(A–N) 3D docking mode diagram of amitriptyline, mirtazapine, and escitalopram with target proteins. Amitriptyline with (A) MAOB, (B) SLC6A4, (C) SCL6A3, (D) DRD2, and (E) CYP2D6. Mirtazapine with (F) SLC6A4, (G) HTR2C, (H) DRD2, (I) CYP2D6, and (J) CHRNA4. Escitalopram with (K) DRD2, (L) HTR2C, (M) SLC6A3, and (N) NR3C1.

**Figure:**
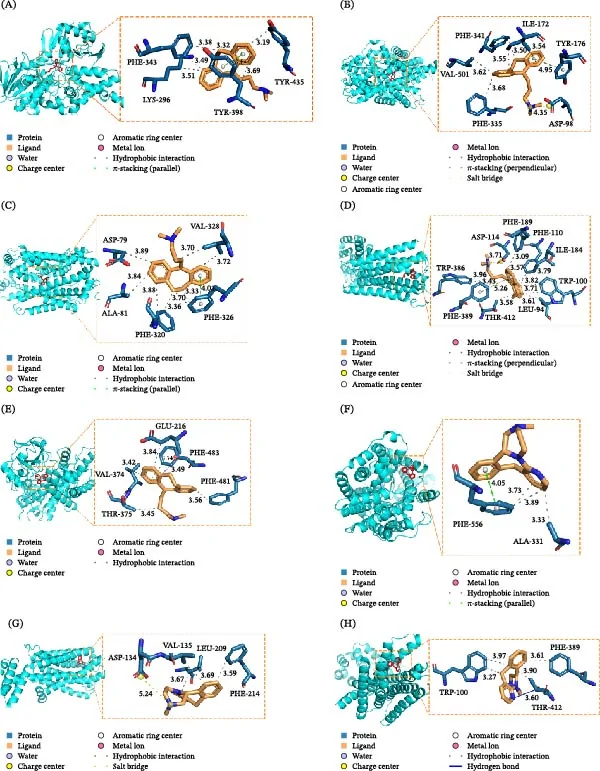

**Figure:**
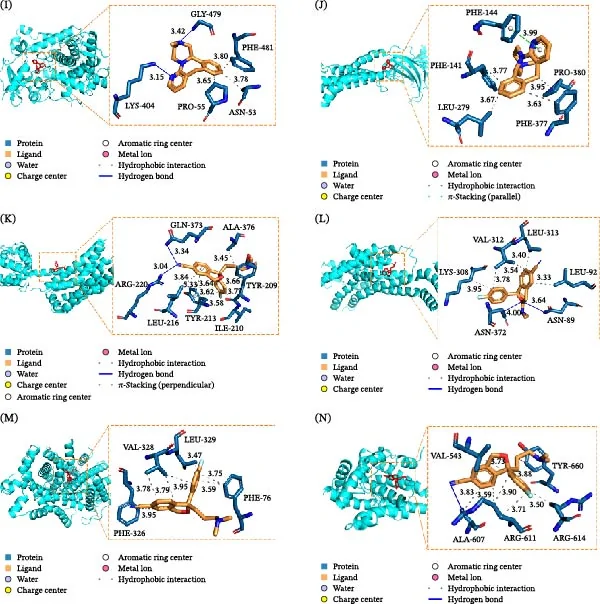

**Table 6:** Molecular interaction of small molecules with target proteins.

Drugs	MAOB	SLC6A4	SLC6A3	DRD2	CYP2D6	HTR2C	CHRNA4	NR3C1
Amitriptyline	^∗∗∗^	^∗∗∗∗∗∗^	^∗∗∗^	^∗∗∗∗∗∗^	^∗^	—	—	—
Mirtazapine	—	^∗∗∗^	—	^∗∗^	^∗∗^	^∗∗∗∗^	^∗∗∗^	—
Escitalopram	—	—	^∗^	^∗∗∗∗∗^	—	^∗∗^	—	^∗∗^

*Note:*  ^∗^: Hydrophobic interaction;  ^∗∗^: hydrogen bond and hydrophobic interaction;  ^∗∗∗^: π‐stacking (parallel) and hydrophobic interaction;  ^∗∗∗∗^: salt bridge and hydrophobic interaction;  ^∗∗∗∗∗^: π‐stacking (perpendicular), hydrogen bond, and hydrophobic interaction; and  ^∗∗∗∗∗∗^: salt bridge, π‐stacking (perpendicular), and hydrophobic interaction.

Mirtazapine interacts with SLC6A4 and CHRNA4 through π‐stacking (parallel) and hydrophobic interactions. Mirtazapine interacts with HTR2C through salt bridge and hydrophobic interactions and interacts with DRD2 and CYP2D6 through hydrogen bond and hydrophobic interactions. These interactions suggest that mirtazapine can bind to different targets through noncovalent interactions, including π‐stacking, salt bridges, and hydrophobic interactions, which generally mediate strong binding abilities. SLC6A4 (serotonin transporter), CHRNA4 (nicotinic acetylcholine receptor subtype), and HTR2C (serotonin receptor subtype) are all key targets involved in the regulation of neurotransmission. The antidepressant effects of mirtazapine are closely related to the functional modulation of these targets. Escitalopram interacts with DRD2 through π‐stacking (perpendicular), hydrogen bond, and hydrophobic interactions and interacts with HTR2C and NR3C1 through hydrogen bond and hydrophobic interactions. These targets are involved in serotonin, dopamine, and glucocorticoid signaling pathways. These interactions suggest that escitalopram can bind to multiple neurotransmitter and hormone receptors regulating the serotonin system, dopamine system, and stress response through noncovalent interactions. Thus, the antidepressant effects of escitalopram may be related to the modulation of multiple neurotransmission and stress regulation mechanisms.

## 4. Discussion

Cipriani and colleagues conducted a comprehensive evaluation of 21 commonly used antidepressants for the acute treatment of MDD in adults, based on the best available evidence. The study assessed the efficacy (measured by remission rates) and tolerability (measured by dropout rates) of these medications. Their analyses found that amitriptyline, mirtazapine, and escitalopram were among the most effective drugs for treating MDD, while fluoxetine and trazodone ranked lower [4]. In our study, we further explored the mechanisms of action of these three most effective antidepressants using NP and molecular docking techniques. Venn diagram analysis revealed both overlapping and distinct targets for amitriptyline, mirtazapine, and escitalopram in the treatment of MDD. A total of 48 common targets were identified, which were likely crucial for the antidepressant effects of these drugs. These common targets primarily involved pathways closely related to antidepressant activity, including serotonergic (5‐HT) and dopaminergic pathways. Antidepressants work by inhibiting serotonin reuptake or directly acting on these receptors, thereby increasing serotonin concentration in the synaptic cleft [44]. Additionally, antidepressants like amitriptyline regulate mood by modulating dopamine receptors (DRD1, DRD2, DRD3, DRD4, and DRD5), which play key roles in motivation, pleasure, and reward systems. Monoamine transporters responsible for the reuptake of NE (SLC6A2), dopamine (SLC6A3), and serotonin (SLC6A4) are also key targets for antidepressant action. TCAs, such as amitriptyline, primarily enhance NE and serotonin neurotransmission by inhibiting the reuptake of SLC6A2 and SLC6A4. This multitarget mechanism likely contributes to the higher efficacy of these drugs in the treatment of MDD.

### 4.1. Common Targets and Shared Mechanisms

The common targets of the three drugs are also associated with the side effects of antidepressants. For instance, blocking adrenergic receptors (ADRA1A, ADRA1B, ADRA1D, ADRA2A, ADRA2B, and ADRA2C) may result in hypotension, dizziness, and bradycardia [45]. Excessive activation of dopamine receptors can lead to motor disturbances, tremors, anxiety, and agitation [46]. Long‐term use of these drugs, particularly TCAs such as amitriptyline or antipsychotics, may result in drug‐induced extrapyramidal symptoms. Some antidepressants, such as mirtazapine, have sedative and anxiolytic effects; however, drowsiness, weight gain, and increased appetite are caused by the blockage of HRH1 receptors [47, 48]. Overactivation of postsynaptic serotonin receptors can result in sexual dysfunction, weight gain, nausea, anxiety, and insomnia [44]. Specifically, HTR2C is linked to weight gain, while HTR3A is associated with nausea and vomiting. Inhibition of monoamine transporters, such as SLC6A4, may also lead to sexual dysfunction, including reduced libido and erectile dysfunction. MAPK14 is involved in cell proliferation and inflammation, and antidepressants may regulate this pathway to reduce depression‐related neuroinflammation [49, 50].

### 4.2. Functional Roles of Core Targets in Depression Pathophysiology and Clinical Relevance

To bridge the gap between molecular mechanisms and clinical applications, we further analyzed the depression‐related functions of four core targets (DRD2, CYP2D6, MAOB, and SLC6A4).

Dopamine D2 receptor–targeting agents are widely used to treat neuropsychiatric disorders, including depression. In depression, impaired DRD2 signaling is closely linked with anhedonia and psychomotor retardation—two hallmark symptoms. A neuroimaging study shows that sustained D2/D3 receptor blockade in healthy volunteers induces these negative symptoms, which are correlated with reduced striatal responses to reward cues [51]. Thus, targeting DRD2 may help alleviate reward‐related deficits in depression.

As a major drug‐metabolizing enzyme, CYP2D6 plays a critical role in antidepressant metabolism. A large prospective cohort study of 921 patients demonstrates that poor efficacy of paroxetine is associated with ultrarapid metabolizer status, while lower CYP2D6 activity scores are correlated with a higher risk of adverse reactions [52]. These findings highlight the clinical value of CYP2D6 metabolic phenotype for personalized antidepressant therapy. Elevated MAOB distribution volume, an index of MAOB density, is reported in the prefrontal cortex of patients with major depressive episodes [53]. Increased MAOB activity reduces dopamine availability and increases oxidative stress—changes that are poorly addressed by standard SSRIs. Thus, MAOB inhibition may benefit patients with depression who exhibit anhedonia or other dopamine‐related symptoms, supporting the use of MAOB inhibitors as adjunctive treatment for SSRI‐resistant depression.

The serotonin transporter (5‐HTT), encoded by SLC6A4, terminates serotonergic signaling by recycling serotonin from the synaptic space into presynaptic neurons [54]. Many antidepressants (e.g., SSRIs and SNRIs) act by binding to 5‐HTT and inhibiting this reuptake process. Carriers of the serotonin‐transporter‐linked polymorphic region (5‐HTTLPR) long (L) allele show better antidepressant response and fewer adverse reactions, particularly with SSRIs and in European populations [55], suggesting that 5‐HTTLPR genotyping may guide personalized antidepressant treatment.

### 4.3. Drug‐Specific Mechanisms and Key Targets

#### 4.3.1. Amitriptyline

Screening results of the core targets indicate that the key targets mediating the effects of the three drugs on MDD differ. In addition to acting on monoamine receptors, amitriptyline interacts with CYP450 enzymes (such as CYP3A4, CYP2C19, CYP1A2, CYP2C9, and CYP3A5), thereby affecting the metabolism of drugs by these enzymes. This interaction may lead to increased plasma concentrations of amitriptyline, which can cause side effects such as drowsiness, dry mouth, or arrhythmias. Due to the significant polymorphism of CYP450 enzymes [56], individuals with high CYP2D6 activity (ultrarapid metabolizers) may clear the drug too quickly, reducing its therapeutic efficacy [57]. Amitriptyline is metabolized by CYP2C19 into its active metabolite nortriptyline, which is also a pharmacologically active TCA.

#### 4.3.2. Mirtazapine

Unlike amitriptyline and escitalopram, the key targets of mirtazapine are monoamine transporters (SLC6A3, SLC6A4, and SLC6A2) and serotonin receptors (HTR1A, HTR2A, HTR2C, and HTR3A). This finding supports the known mechanism of action of mirtazapine. Mirtazapine is an α2‐adrenergic receptor antagonist, which has an indirect effect on NE and 5‐HT levels in the brain [58]. By inhibiting α2 autoreceptors, mirtazapine increases NE release, which contributes to improved sleep [59]. Unlike other SSRIs, mirtazapine enhances sleep efficiency and continuity without suppressing rapid eye movement (REM) sleep [60]. Additionally, by inhibiting α2 heteroreceptors, mirtazapine further promotes serotonin release in the brain. Mirtazapine also antagonizes HTR2 and HTR3 receptors; HTR2 antagonists help alleviate serotonin‐related anxiety symptoms, while HTR3 antagonists help prevent side effects like nausea and gastrointestinal side effects [61, 62]. As a result, mirtazapine enhances the transmission of serotonin in mood‐improving pathways while avoiding some of the negative side effects associated with other serotonin receptor subtypes, such as sexual dysfunction or nausea [59]. This dual mechanism may contribute to mirtazapine’s antidepressant and anxiolytic properties.

#### 4.3.3. Escitalopram

The primary targets of escitalopram include serotonin (5‐HT) receptors (HTR2A, HTR1A, HTR1B, HTR2C, and HTR3A), monoamine transporters (SLC6A4), and SRC. Escitalopram inhibits SLC6A4, leading to an increase in serotonin levels in the synaptic cleft [63]. This elevation in synaptic serotonin desensitizes postsynaptic HTR2A and HTR1A receptors [64], which results in a reduction in anxiety and depression in patients with MDD [65, 66]. Additionally, the rise in serotonin levels activates HTR1B receptors, reducing aggression and impulsivity in depressive patients. The high polymorphism in the SLC6A4 gene affects the efficacy of escitalopram, posing challenges for personalized clinical treatment [67]. Escitalopram indirectly modulates HTR2C and HTR3A receptors. Desensitization of HTR2C may improve anxiety symptoms and appetite, potentially leading to weight gain [68]. Modulating HTR3A‐related pathways can reduce common SSRI side effects, including nausea and gastrointestinal disturbances [69]. SRC is involved in intracellular pathways related to cell growth, survival, and differentiation, which are crucial for brain development and neuroplasticity. Escitalopram’s interaction with SRC may improve cognitive functions such as memory and attention in MDD patients, although specific evidence supporting SRC’s role in escitalopram‐mediated effects on MDD is lacking. These findings underscore the importance of target differences among these drugs in treating MDD and provide new insights for personalized treatment strategies.

### 4.4. Pathway Enrichment Analysis

The enrichment analysis indicated that the antidepressant effects of the three drugs involve GPCRs, and the key 5‐HT receptors are all GPCRs, highlighting their crucial role in regulating mood and alleviating depressive symptoms. The three antidepressants influence synaptic transmission, primarily by increasing neurotransmitter levels (5‐HT and NE) in the synaptic cleft. For example, escitalopram increases serotonin levels by inhibiting the SLC6A4‐mediated serotonin reuptake. Amitriptyline affects both serotonin and NE, and mirtazapine enhances NE and serotonin release by antagonizing α2 receptors. The “neuroactive ligand–receptor interaction” pathway, which plays a key role in transmitting signals between neurons via neurotransmitters, is central to the functions of all three drugs. However, the mechanisms of action of the three drugs differ. Escitalopram affects 5‐HT receptors, especially HTR3A. The highly selective mechanism of action of escitalopram results in reduced SSRI side effects. Thus, escitalopram is a first‐line treatment for anxiety and depression. In contrast, amitriptyline has broader effects, including effects on calcium signaling, which contribute to the effects of amitriptyline on complex depressive symptoms. However, the broad effects of amitriptyline result in more side effects. Amitriptyline antagonizes histamine H1 and α1‐adrenergic receptors, leading to sedation and weight gain. Mirtazapine blocks α2‐adrenergic receptors, leading to more NE release and faster sleep improvements compared with the other two drugs [70]. Mirtazapine improves sleep efficiency and continuity, and, in contrast to other SSRIs, mirtazapine does not suppress REM sleep. Mirtazapine induces both serotonin and NE release, striking a balance between efficacy and fewer serotonin‐related side effects [71].

Enrichment analysis indicates that the antidepressant effects of all three drugs involve GPCRs, with key 5‐HT receptors being GPCRs [72, 73], highlighting their critical role in mood regulation and alleviation of depressive symptoms. As described, these antidepressants intervene in synaptic transmission primarily by affecting neurotransmitter levels in the synaptic cleft (serotonin and NE). The “Neuroactive Ligand–Receptor Interaction” pathway plays a crucial role in neurotransmitter signaling between neurons and is central to the function of all three drugs [45, 74]. However, the mechanisms of these drugs differ. Escitalopram primarily affects 5‐HT receptors, particularly HTR3A, with its highly selective action reducing SSRI‐associated side effects, making escitalopram a first‐line treatment for anxiety and depression [4, 75]. In contrast, amitriptyline has a broader mechanism of action, including effects on calcium signaling, which contribute to its impact on complex depressive symptoms. Amitriptyline antagonizes histamine H1 and α1‐adrenergic receptors, leading to sedation and weight gain.

### 4.5. Molecular Docking Results

The molecular docking results revealed similarities in how the three drugs bind to their targets. All three drugs show good affinity for SLC6A4, suggesting that their antidepressant effects are largely driven by regulating serotonin reuptake, thereby increasing serotonin concentration in the synaptic cleft. The binding interactions involve π‐stacking, hydrogen bonds, and hydrophobic interactions, which contribute to the stability of the drug‐target complexes. Stable binding enhances both affinity and efficacy. However, the docking results also showed differences among the three drugs. Amitriptyline exhibited stronger affinity for MAOB and DRD2, highlighting its dual role in regulating dopamine and serotonin transport, which supports its potent antidepressant effects [76]. Amitriptyline, mirtazapine, and escitalopram may indirectly influence MAO activity through their modulation of neurotransmitters such as serotonin, dopamine, and NE. On the one hand, the broad action of amitriptyline contributes to its more frequent side effects. Mirtazapine, on the other hand, targets both 5‐HT receptors and the cholinergic system [8, 45], with a relatively balanced affinity. This suggests that mirtazapine’s broad antidepressant mechanism may benefit different types of patients. Compared with the other two drugs, escitalopram’s target affinity was slightly lower. However, it binds effectively to serotonin system regulators, such as SLC6A4 and HTR2C, supporting its function as an SSRI. Additionally, escitalopram’s interaction with the glucocorticoid receptor NR3C1 is associated with its role in stress regulation, mood stabilization, and anxiety relief [77], extending escitalopram’s effects beyond the serotonin system.

### 4.6. Clinical Implications and Personalized Antidepressant Therapy

This study explored the key targets and mechanisms of action of three antidepressants: amitriptyline, mirtazapine, and escitalopram. These drugs reflect distinct antidepressant mechanisms through different targets and interactions. Amitriptyline demonstrates broader and stronger target binding, making it more effective for patients with more severe depression, but it also causes more side effects [78]. Mirtazapine has a wider range of target distribution and fewer side effects [79], potentially making it effective for a broader range of patients. Escitalopram, as an SSRI, is well‐suited for patients with serotonin system dysfunction and has better tolerability compared to the other two drugs [80]. The molecular docking results in this study highlight the differences in target binding, providing valuable insights for further drug optimization. As a result, combining different antidepressants can enhance therapeutic effects while balancing potential side effects [81]. For example, the well‐known “California rocket fuel” combination of mirtazapine (an α2 antagonist and 5‐HT2/5‐HT3 blocker) with venlafaxine (a serotonin‐NE reuptake inhibitor) has shown favorable outcomes for treatment‐resistant depression [82–84]. Given the slow progress in developing new antidepressants, future research should focus on exploring targets to develop more precise antidepressants. Emphasizing the clinical application of existing effective drugs can quickly benefit patients with MDD. Future drug development should aim to produce medications with both high efficacy and better tolerability. Additionally, exploring more individualized treatment approaches based on patient differences is a viable direction for future studies.

## 5. Limitations

This study provides valuable insights into the similarities and differences in the antidepressant mechanisms of amitriptyline, mirtazapine, and escitalopram. However, the study has several limitations. The computational biology methods used in the study rely on existing data resources and may overlook the complexity of drug interactions in vivo. Future studies should refine experimental validation through in vitro or in vivo research to confirm the computational predictions of this study and ensure clinical relevance. While focusing on key targets and pathways is important, the potential influence of other targets and mechanisms on antidepressant effects cannot be ruled out. Molecular docking reveals binding affinities between drugs and their targets but does not account for dynamic interactions over time, protein flexibility, or potential in vivo conformational changes. Antidepressant treatments require extended treatment; thus, factors such as patient willingness, tolerance to side effects, personality traits, financial situations, and family support also impact the effectiveness of treatment. This study considered the genetic specificity of metabolic enzymes and receptor polymorphisms but did not include them in the research due to the limitations of the study’s scope. Additionally, environmental, psychological, and comorbid factors are not sufficiently reflected in computational models, which is a common limitation of such studies.

## 6. Conclusions

The similarities and differences in antidepressant effects and side‐effect mechanisms of amitriptyline, mirtazapine, and escitalopram were explored using NP. Online databases were leveraged to identify overlapping and independent targets for the three drugs in the treatment of MDD. The targets primarily involved “chemical synaptic transmission” and “G protein‐coupled receptor signaling pathway.“ Key pathways, including “Neuroactive ligand‐receptor interaction,” “Calcium signaling pathway,” and “Serotonergic synapse,” played crucial roles. Molecular docking indicates that specific binding of amitriptyline, mirtazapine, and escitalopram to multiple targets, including MAOB, SLC6A4, DRD2, HTR2C, and SLC6A3, contributes to their antidepressant effects and potential side effects. These drugs share common mechanisms of action, such as the regulation of 5‐HT, NE, and dopamine. However, the drugs differ in target specificity, pathways, and binding modes. Amitriptyline exhibits stronger antidepressant effects and broader binding sites, making it suitable for complex MDD cases but also causing more side effects like drowsiness, dry mouth, and arrhythmia. Individual variations in metabolism also impact the efficacy of amitriptyline. The effects of mirtazapine are elicited through monoamine transporters and 5‐HT receptors. Thus, mirtazapine evokes broad target modulation, reliable efficacy, and fewer serotonin‐related side effects, such as gastrointestinal issues and sexual dysfunction. The unique blockade of α2‐adrenergic receptors by mirtazapine improves sleep, supporting its clinical use as a first‐line treatment for depression and anxiety. Escitalopram specifically targets 5‐HT‐related receptors and has a narrower scope than amitriptyline, with fewer side effects. Escitalopram may also improve attention and memory. These findings provide important evidence for the first‐line use of these drugs in treating MDD and support the development of personalized treatment strategies.

## Author Contributions

Qiuyu Zhang contributed to the acquisition and interpretation of data, and drafted the manuscript. Chuanjun Zhuo contributed to the conception and design of the work, interpretation of data, drafting of the manuscript, and critical revision for important intellectual content. Haitao Song, Jiatong Zou, Ranli Li, Xiaoyan Ma, Ximing Chen, Yachen Li, Lei Yang, Lina Wang, Chao Li, and Hongjun Tian contributed to the acquisition and interpretation of data and critically revised the manuscript for important intellectual content.

## Funding

This work was supported by grants from the National Natural Science Foundation of China (Grants 82171503 and 81871052) awarded to Chuanjun Zhuo.

## Disclosure

All authors have read and approved the final manuscript.

## Conflicts of Interest

The authors declare no conflicts of interest.

## Data Availability

The data that support the findings of this study are available from the corresponding author upon reasonable request.

## References

[1] GBD Mental Disorders Collaborators , Global, Regional, and National Burden of 12 Mental Disorders in 204 Countries and Territories, 1990–2019: A Systematic Analysis for the Global Burden of Disease Study 2019, Lancet Psychiatry. (2022) 9, no. 2, 137–150, 10.1016/S2215-0366(21)00395-3. PMC8776563 35026139

[2] GBD 2021 Diseases and Injuries Collaborators , Global Incidence, Prevalence, Years Lived With Disability (YLDs), Disability-Adjusted Life-Years (DALYs), and Healthy Life Expectancy (HALE) for 371 Diseases and Injuries in 204 Countries and Territories and 811 Subnational Locations, 1990–2021: A Systematic Analysis for the Global Burden of Disease Study 2021, The Lancet. (2024) 403, no. 10440, 2133–2161, 10.1016/S0140-6736(24)00757-8. PMC11122111 38642570

[3] García-Velázquez R. , Jokela M. , and Rosenström T. H. , The Varying Burden of Depressive Symptoms Across Adulthood: Results From Six NHANES Cohorts, Journal of Affective Disorders. (2019) 246, 290–299, 10.1016/j.jad.2018.12.059. 30594042

[4] Cipriani A. , Furukawa T. A. , and Salanti G. , et al.Comparative Efficacy and Acceptability of 21 Antidepressant Drugs for the Acute Treatment of Adults With Major Depressive Disorder: A Systematic Review and Network Meta-Analysis, Lancet. (2018) 391, no. 10128, 1357–1366, 10.1016/S0140-6736(17)32802-7. PMC5889788 29477251

[5] Larrey D. , Amouyal G. , and Pessayre D. , et al.Amitriptyline-Induced Prolonged Cholestasis, Gastroenterology. (1988) 94, no. 1, 200–203, 10.1016/0016-5085(88)90631-2. 3335290

[6] Scollins M. J. , Robinson D. S. , and Nies A. , Cardiotoxicity of Amitriptyline, The Lancet. (1972) 300, no. 7788, 10.1016/S0140-6736(72)92631-1, 1202. 4117629

[7] Brueckle M. S. , Thomas E. T. , and Seide S. E. , et al.Amitriptyline’s Anticholinergic Adverse Drug Reactions—A Systematic Multiple-Indication Review and Meta-Analysis, PLoS ONE. (2023) 18, no. 4, 10.1371/journal.pone.0284168, e0284168. PMC10075391 37018325

[8] Anttila S. A. K. and Leinonen E. V. J. , A Review of the Pharmacological and Clinical Profile of Mirtazapine, CNS Drug Reviews. (2001) 7, no. 3, 249–264, 10.1111/j.1527-3458.2001.tb00198.x. PMC6494141 11607047

[9] Watanabe N. , Omori I. M. , and Nakagawa A. , et al.Mirtazapine Versus Other Antidepressive Agents for Depression, Cochrane Database of Systematic Reviews. (2011) 2011, no. 12, 10.1002/14651858.CD006528.pub2, CD006528. PMC4158430 22161405

[10] Cipriani A. , Santilli C. , and Furukawa T. A. , et al.Escitalopram Versus Other Antidepressive Agents for Depression, Cochrane Database of Systematic Reviews. (2016) 2016, no. 5, 10.1002/14651858.CD006532.pub2, CD006532. PMC4164382 19370639

[11] Baldwin D. S. , Reines E. H. , Guiton C. , and Weiller E. , Escitalopram Therapy for Major Depression and Anxiety Disorders, Annals of Pharmacotherapy. (2007) 41, no. 10, 1583–1592, 10.1345/aph.1K089. 17848424

[12] Warden D. , Rush A. J. , Trivedi M. H. , Fava M. , and Wisniewski S. R. , The STAR∗D Project Results: A Comprehensive Review of Findings, Current Psychiatry Reports. (2007) 9, no. 6, 449–459, 10.1007/s11920-007-0061-3. 18221624

[13] Li X. , Qin X.-M. , Tian J.-S. , Gao X.-X. , Du G.-H. , and Zhou Y.-Z. , Integrated Network Pharmacology and Metabolomics to Dissect the Combination Mechanisms of *Bupleurum Chinense* DC-*Paeonia lactiflora* Pall Herb Pair for Treating Depression, Journal of Ethnopharmacology. (2021) 264, 10.1016/j.jep.2020.113281, 113281. 32810624

[14] Zhao Q. , Pan W. , and Shi H. , et al.Network Pharmacology and Molecular Docking Analysis on the Mechanism of Baihe Zhimu Decoction in the Treatment of Postpartum Depression, Medicine. (2022) 101, no. 43, 10.1097/MD.0000000000029323, e29323. PMC9622608 36316904

[15] Guo X. , Su L. , and Shi M. , et al.Network Pharmacology and Transcriptomics to Explore the Pharmacological Mechanisms of 20(S)-Protopanaxatriol in the Treatment of Depression, International Journal of Molecular Sciences. (2024) 25, no. 14, 10.3390/ijms25147574, 7574. PMC11276827 39062817

[16] Li C. , Tian H. , and Li R. , et al.Molecular Mechanisms of Quetiapine Bidirectional Regulation of Bipolar Depression and Mania Based on Network Pharmacology and Molecular Docking: Evidence From Computational Biology, Journal of Affective Disorders. (2024) 355, 528–539, 10.1016/j.jad.2024.03.096. 38518857

[17] Hopkins A. L. , Network Pharmacology: The Next Paradigm in Drug Discovery, Nature Chemical Biology. (2008) 4, no. 11, 682–690, 10.1038/nchembio.118. 18936753

[18] Zhang P. , Zhang D. , and Zhou W. , et al.Network Pharmacology: Towards the Artificial Intelligence-Based Precision Traditional Chinese Medicine, Briefings in Bioinformatics. (2024) 25, no. 1, 10.1093/bib/bbad518, bbad518. PMC10777171 38197310

[19] Nogales C. , Mamdouh Z. M. , List M. , Kiel C. , Casas A. I. , and Schmidt H. H. H. W. , Network Pharmacology: Curing Causal Mechanisms Instead of Treating Symptoms, Trends in Pharmacological Sciences. (2022) 43, no. 2, 136–150, 10.1016/j.tips.2021.11.004. 34895945

[20] Wishart D. S. , Knox C. , and Guo A. C. , et al.DrugBank: A Comprehensive Resource for in Silico Drug Discovery and Exploration, Nucleic Acids Research. (2006) 34, no. 90001, D668–D672, 10.1093/nar/gkj067. PMC1347430 16381955

[21] Zhou Y. , Zhang Y. , and Zhao D. , et al.TTD: *Therapeutic Target Database* Describing Target Druggability Information, Nucleic Acids Research. (2024) 52, no. D1, D1465–D1477, 10.1093/nar/gkad751. PMC10767903 37713619

[22] Ru J. , Li P. , and Wang J. , et al.TCMSP: A Database of Systems Pharmacology for Drug Discovery From Herbal Medicines, Journal of Cheminformatics. (2014) 6, no. 1, 10.1186/1758-2946-6-13, 13. PMC4001360 24735618

[23] UniProt Consortium , UniProt: The Universal Protein Knowledgebase in 2023, Nucleic Acids Research. (2023) 51, no. D1, D523–D531, 10.1093/nar/gkac1052. PMC9825514 36408920

[24] Freshour S. L. , Kiwala S. , and Cotto K. C. , et al.Integration of the Drug-Gene Interaction Database (DGIdb 4.0) With Open Crowdsource Efforts, Nucleic Acids Research. (2021) 49, no. D1, D1144–D1151, 10.1093/nar/gkaa1084. PMC7778926 33237278

[25] Yao Z.-J. , Dong J. , and Che Y.-J. , et al.TargetNet: A Web Service for Predicting Potential Drug-Target Interaction Profiling via Multi-Target SAR Models, Journal of Computer-Aided Molecular Design. (2016) 30, no. 5, 413–424, 10.1007/s10822-016-9915-2. 27167132

[26] Kim S. , Chen J. , and Cheng T. , et al.PubChem 2023 Update, Nucleic Acids Research. (2023) 51, no. D1, D1373–D1380, 10.1093/nar/gkac956. PMC9825602 36305812

[27] Daina A. , Michielin O. , and Zoete V. , SwissTargetPrediction: Updated Data and New Features for Efficient Prediction of Protein Targets of Small Molecules, Nucleic Acids Research. (2019) 47, no. W1, W357–W364, 10.1093/nar/gkz382. PMC6602486 31106366

[28] Stelzer G. , Rosen N. , and Plaschkes I. , et al.The GeneCards Suite: From Gene Data Mining to Disease Genome Sequence Analyses, Current Protocols in Bioinformatics. (2016) 54, no. 1, 1.30.1–1.30.33, 10.1002/cpbi.5. 27322403

[29] Piñero J. , Ramírez-Anguita J. M. , and Saüch-Pitarch J. , et al.The DisGeNET Knowledge Platform for Disease Genomics: 2019 Update, Nucleic Acids Research. (2019) 48, no. D1, D845–D855, 10.1093/nar/gkz1021. PMC7145631 31680165

[30] Amberger J. S. , Bocchini C. A. , Schiettecatte F. , Scott A. F. , and Hamosh A. , OMIM.org: Online Mendelian Inheritance in Man (OMIM), an Online Catalog of Human Genes and Genetic Disorders, Nucleic Acids Research. (2015) 43, no. D1, D789–D798, 10.1093/nar/gku1205. PMC4383985 25428349

[31] Whirl-Carrillo M. , Huddart R. , and Gong L. , et al.An Evidence-Based Framework for Evaluating Pharmacogenomics Knowledge for Personalized Medicine, Clinical Pharmacology & Therapeutics. (2021) 110, no. 3, 563–572, 10.1002/cpt.2350. PMC8457105 34216021

[32] Szklarczyk D. , Kirsch R. , and Koutrouli M. , et al.The STRING Database in 2023: Protein-Protein Association Networks and Functional Enrichment Analyses for Any Sequenced Genome of Interest, Nucleic Acids Research. (2023) 51, no. D1, D638–D646, 10.1093/nar/gkac1000. PMC9825434 36370105

[33] Shannon P. , Markiel A. , and Ozier O. , et al.Cytoscape: A Software Environment for Integrated Models of Biomolecular Interaction Networks, Genome Research. (2003) 13, no. 11, 2498–2504, 10.1101/gr.1239303. PMC403769 14597658

[34] Jeong H. , Mason S. P. , Barabási A.-L. , and Oltvai Z. N. , Lethality and Centrality in Protein Networks, Nature. (2001) 411, no. 6833, 41–42, 10.1038/35075138. 11333967

[35] Karim M. R. , Morshed M. N. , and Iqbal S. , et al.A Network Pharmacology and Molecular-Docking-Based Approach to Identify the Probable Targets of Short-Chain Fatty-Acid-Producing Microbial Metabolites Against Kidney Cancer and Inflammation, Biomolecules. (2023) 13, no. 11, 10.3390/biom13111678, 1678. PMC10669250 38002360

[36] Newman M. E. J. , A Measure of Betweenness Centrality Based on Random Walks, Social Networks. (2005) 27, no. 1, 39–54, 10.1016/j.socnet.2004.11.009.

[37] Tang Y. , Li M. , Wang J. , Pan Y. , and Wu F.-X. , CytoNCA: A Cytoscape Plugin for Centrality Analysis and Evaluation of Protein Interaction Networks, Biosystems. (2015) 127, 67–72, 10.1016/j.biosystems.2014.11.005. 25451770

[38] Huang D. W. , Sherman B. T. , and Lempicki R. A. , Systematic and Integrative Analysis of Large Gene Lists Using DAVID Bioinformatics Resources, Nature Protocols. (2009) 4, no. 1, 44–57, 10.1038/nprot.2008.211. 19131956

[39] Huang D. W. , Sherman B. T. , and Lempicki R. A. , Bioinformatics Enrichment Tools: Paths Toward the Comprehensive Functional Analysis of Large Gene Lists, Nucleic Acids Research. (2009) 37, no. 1, 1–13, 10.1093/nar/gkn923. PMC2615629 19033363

[40] Chen L. , Zhang Y.-H. , and Wang S. P. , et al.Prediction and Analysis of Essential Genes Using the Enrichments of Gene Ontology and KEGG Pathways, PLoS ONE. (2017) 12, no. 9, 10.1371/journal.pone.0184129, e0184129. PMC5584762 28873455

[41] Trott O. and Olson A. J. , AutoDock Vina: Improving the Speed and Accuracy of Docking With a New Scoring Function, Efficient Optimization, and Multithreading, Journal of Computational Chemistry. (2010) 31, no. 2, 455–461, 10.1002/jcc.21334. PMC3041641 19499576

[42] Berman H. M. , Westbrook J. , and Feng Z. , et al.The Protein Data Bank, Nucleic Acids Research. (2000) 28, no. 1, 235–242, 10.1093/nar/28.1.235. PMC102472 10592235

[43] Adasme M. F. , Linnemann K. L. , and Bolz S. N. , et al.PLIP 2021: Expanding the Scope of the Protein-Ligand Interaction Profiler to DNA and RNA, Nucleic Acids Research. (2021) 49, no. W1, W530–W534, 10.1093/nar/gkab294. PMC8262720 33950214

[44] Blier P. , Pharmacology of Rapid-Onset Antidepressant Treatment Strategies, The Journal of Clinical Psychiatry. (2001) 62, 12–17. 11444761

[45] Millan M. J. , Multi-Target Strategies for the Improved Treatment of Depressive States: Conceptual Foundations and Neuronal Substrates, Drug Discovery and Therapeutic Application, Pharmacology & Therapeutics. (2006) 110, no. 2, 135–370, 10.1016/j.pharmthera.2005.11.006. 16522330

[46] Ashok A. H. , Marques T. R. , and Jauhar S. , et al.The Dopamine Hypothesis of Bipolar Affective Disorder: The State of the Art and Implications for Treatment, Molecular Psychiatry. (2017) 22, no. 5, 666–679, 10.1038/mp.2017.16. PMC5401767 28289283

[47] Schwartz J. C. , Arrang J. M. , Garbarg M. , Pollard H. , and Ruat M. , Histaminergic Transmission in the Mammalian Brain, Physiological Reviews. (1991) 71, no. 1, 1–51, 10.1152/physrev.1991.71.1.1. 1846044

[48] Munari L. , Provensi G. , and Passani M. B. , et al.Brain Histamine Is Crucial for Selective Serotonin Reuptake Inhibitors’ Behavioral and Neurochemical Effects, International Journal of Neuropsychopharmacology. (2015) 18, no. 10, 10.1093/ijnp/pyv045, pyv045. PMC4648163 25899065

[49] Behl T. , Rana T. , and Alotaibi G. H. , et al.Polyphenols Inhibiting MAPK Signalling Pathway Mediated Oxidative Stress and Inflammation in Depression, Biomedicine & Pharmacotherapy. (2022) 146, 10.1016/j.biopha.2021.112545, 112545. 34922112

[50] Wang X.-L. , Yuan K. , Zhang W. , Li S.-X. , Gao G. F. , and Lu L. , Regulation of Circadian Genes by the MAPK Pathway: Implications for Rapid Antidepressant Action, Neuroscience Bulletin. (2020) 36, no. 1, 66–76, 10.1007/s12264-019-00358-9. PMC6940409 30859414

[51] Osugo M. , Wall M. B. , and Selvaggi P. , et al.Striatal Dopamine D2/D3 Receptor Regulation of Human Reward Processing and Behaviour, Nature Communications. (2025) 16, no. 1, 10.1038/s41467-025-56663-7, 1852. PMC11845780 39984436

[52] Liao Y. , Sun Y. , and Guo J. , et al.Dose Adjustment of Paroxetine Based on CYP2D6 Activity Score Inferred Metabolizer Status in Chinese Han Patients With Depressive or Anxiety Disorders: A Prospective Study and Cross-Ethnic Meta-Analysis, eBioMedicine. (2024) 104, 10.1016/j.ebiom.2024.105165, 105165. PMC11141156 38776596

[53] Moriguchi S. , Wilson A. A. , and Miler L. , et al.Monoamine Oxidase B Total Distribution Volume in the Prefrontal Cortex of Major Depressive Disorder: An [11C]SL25.1188 Positron Emission Tomography Study, JAMA Psychiatry. (2019) 76, no. 6, 634–641, 10.1001/jamapsychiatry.2019.0044. PMC6551845 30840042

[54] Bousman C. A. , Stevenson J. M. , and Ramsey L. B. , et al.Clinical Pharmacogenetics Implementation Consortium (CPIC) Guideline for *CYP2D6*, *CYP2C19*, *CYP2B6*, *SLC6A4*, and *HTR2A* Genotypes and Serotonin Reuptake Inhibitor Antidepressants, Clinical Pharmacology & Therapeutics. (2023) 114, no. 1, 51–68, 10.1002/cpt.2903. PMC10564324 37032427

[55] Stein K. , Maruf A. A. , Müller D. J. , Bishop J. R. , and Bousman C. A. , Serotonin Transporter Genetic Variation and Antidepressant Response and Tolerability: A Systematic Review and Meta-Analysis, Journal of Personalized Medicine. (2021) 11, no. 12, 10.3390/jpm11121334, 1334. PMC8707702 34945806

[56] Gaedigk A. , Sangkuhl K. , Whirl-Carrillo M. , Klein T. , and Leeder J. S. , Prediction of CYP2D6 Phenotype From Genotype Across World Populations, Genetics in Medicine. (2017) 19, no. 1, 69–76, 10.1038/gim.2016.80. PMC5292679 27388693

[57] Dean L. and Kane M. , Pratt V. M. , Scott S. A. , Pirmohamed M. , Esquivel B. , Kattman B. L. , and Malheiro A. J. , Amitriptyline Therapy and CYP2D6 and CYP2C19 Genotype, MINI Medical Genetics Summaries, 2017, National Center for Biotechnology Information (US). 28520380

[58] Hassanein E. H. M. , Althagafy H. S. , Baraka M. A. , Abd-Alhameed E. K. , and Ibrahim I. M. , Pharmacological Update of Mirtazapine: A Narrative Literature Review, Naunyn-Schmiedeberg’s Archives of Pharmacology. (2024) 397, no. 5, 2603–2619, 10.1007/s00210-023-02818-6. PMC11074035 37943296

[59] Coutens B. , Yrondi A. , Rampon C. , and Guiard B. P. , Psychopharmacological Properties and Therapeutic Profile of the Antidepressant Venlafaxine, Psychopharmacology. (2022) 239, no. 9, 2735–2752, 10.1007/s00213-022-06203-8. 35947166

[60] Shen J. , Chung S. A. , and Kayumov L. , et al.Polysomnographic and Symptomatological Analyses of Major Depressive Disorder Patients Treated With Mirtazapine, The Canadian Journal of Psychiatry. (2006) 51, no. 1, 27–34, 10.1177/070674370605100106. 16491981

[61] Timmer C. J. , Sitsen J. M. , and Delbressine L. P. , Clinical Pharmacokinetics of Mirtazapine, Clinical Pharmacokinetics. (2000) 38, no. 6, 461–474, 10.2165/00003088-200038060-00001. 10885584

[62] Davis R. and Wilde M. I. , Mirtazapine: A Review of Its Pharmacology and Therapeutic Potential in the Management of Major Depression, CNS Drugs. (1996) 5, no. 5, 389–402, 10.2165/00023210-199605050-00007. 26071050

[63] Hamon M. and Blier P. , Monoamine Neurocircuitry in Depression and Strategies for New Treatments, Progress in Neuro-Psychopharmacology and Biological Psychiatry. (2013) 45, 54–63, 10.1016/j.pnpbp.2013.04.009. 23602950

[64] Blier P. and Ward N. M. , Is There a Role for 5-HT1A Agonists in the Treatment of Depression?, Biological Psychiatry. (2003) 53, no. 3, 193–203, 10.1016/S0006-3223(02)01643-8. 12559651

[65] Stahl S. M. , Mechanism of Action of Serotonin Selective Reuptake Inhibitors. Serotonin Receptors and Pathways Mediate Therapeutic Effects and Side Effects, Journal of Affective Disorders. (1998) 51, no. 3, 215–235, 10.1016/S0165-0327(98)00221-3. 10333979

[66] Carine B. , Philippe M. , and Joël B. , Choi S. , 5-Hydroxytryptamine Receptor 2C, Encyclopedia of Signaling Molecules, 2018, Springer International Publishing, 24–35.

[67] Holsboer F. , How Can We Realize the Promise of Personalized Antidepressant Medicines?, Nature Reviews Neuroscience. (2008) 9, no. 8, 638–646, 10.1038/nrn2453. 18628772

[68] Quilter C. R. , Bagga M. , Moinie A. , Junaid F. , and Sargent C. A. , Gene Structure and Expression of Serotonin Receptor HTR2C in Hypothalamic Samples From Infanticidal and Control Sows, BMC Neuroscience. (2012) 13, no. 1, 10.1186/1471-2202-13-37, 37. PMC3350401 22471812

[69] Spiller R. and Garsed K. , Postinfectious Irritable Bowel Syndrome, Gastroenterology. (2009) 136, no. 6, 1979–1988, 10.1053/j.gastro.2009.02.074. 19457422

[70] Wichniak A. , Wierzbicka A. , Walęcka M. , and Jernajczyk W. , Effects of Antidepressants on Sleep, Current Psychiatry Reports. (2017) 19, no. 9, 10.1007/s11920-017-0816-4, 63. PMC5548844 28791566

[71] Croom K. F. , Perry C. M. , and Plosker G. L. , Mirtazapine: A Review of Its Use in Major Depression and Other Psychiatric Disorders, CNS Drugs. (2009) 23, no. 5, 427–452, 10.2165/00023210-200923050-00006. 19453203

[72] Barnes N. M. and Sharp T. , A Review of Central 5-HT Receptors and Their Function, Neuropharmacology. (1999) 38, no. 8, 1083–1152, 10.1016/S0028-3908(99)00010-6. 10462127

[73] Carhart-Harris R. L. and Nutt D. J. , Serotonin and Brain Function: A Tale of Two Receptors, Journal of Psychopharmacology. (2017) 31, no. 9, 1091–1120, 10.1177/0269881117725915. PMC5606297 28858536

[74] Yu Y. , Li X. , and Teng T. , et al.Comparative Analysis of the Nucleus Accumbens Transcriptional Features in Multiple Depressive Animal Models, Behavioural Brain Research. (2024) 463, 10.1016/j.bbr.2024.114890, 114890. 38309372

[75] Kennedy S. H. , Andersen H. F. , and Thase M. E. , Escitalopram in the Treatment of Major Depressive Disorder: A Meta-Analysis, Current Medical Research and Opinion. (2009) 25, no. 1, 161–175, 10.1185/03007990802622726. 19210149

[76] Baldessarini R. J. , Current Status of Antidepressants: Clinical Pharmacology and Therapy, The Journal of Clinical Psychiatry. (1989) 50, no. 4, 117–126. 2647712

[77] Klok M. D. , Vreeburg S. A. , Penninx B. W. J. H. , Zitman F. G. , de Kloet E. R. , and DeRijk R. H. , Common Functional Mineralocorticoid Receptor Polymorphisms Modulate the Cortisol Awakening Response: Interaction With SSRIs, Psychoneuroendocrinology. (2011) 36, no. 4, 484–494, 10.1016/j.psyneuen.2010.07.024. 20884124

[78] Gillman P. K. , Tricyclic Antidepressant Pharmacology and Therapeutic Drug Interactions Updated, British Journal of Pharmacology. (2007) 151, no. 6, 737–748, 10.1038/sj.bjp.0707253. PMC2014120 17471183

[79] Fawcett J. and Barkin R. L. , Review of the Results From Clinical Studies on the Efficacy, Safety and Tolerability of Mirtazapine for the Treatment of Patients With Major Depression, Journal of Affective Disorders. (1998) 51, no. 3, 267–285, 10.1016/S0165-0327(98)00224-9. 10333982

[80] Yin J. , Song X. , Wang C. , Lin X. , and Miao M. , Escitalopram Versus Other Antidepressive Agents for Major Depressive Disorder: A Systematic Review and Meta-Analysis, BMC Psychiatry. (2023) 23, no. 1, 10.1186/s12888-023-05382-8, 876. PMC10675869 38001423

[81] Stephen M. S. and Stephen M. S. , Essential Psychopharmacology: Neuroscientific Basis and Practical Applications, 2000, Cambridge University Press.

[82] Hannan N. , Hamzah Z. , Akinpeloye H. O. , and Meagher D. , Venlafaxine—Mirtazapine Combination in the Treatment of Persistent Depressive Illness, Journal of Psychopharmacology. (2007) 21, no. 2, 161–164, 10.1177/0269881107065738. 17329295

[83] Blier P. , Ward H. E. , Tremblay P. , Laberge L. , Hébert C. , and Bergeron R. , Combination of Antidepressant Medications From Treatment Initiation for Major Depressive Disorder: A Double-Blind Randomized Study, American Journal of Psychiatry. (2010) 167, no. 3, 281–288, 10.1176/appi.ajp.2009.09020186. 20008946

[84] Gawliński D. , Smaga I. , Zaniewska M. , Gawlińska K. , Faron-Górecka A. , and Filip M. , Adaptive Mechanisms Following Antidepressant Drugs: Focus on Serotonin 5-HT2A Receptors, Pharmacological Reports. (2019) 71, no. 6, 994–1000, 10.1016/j.pharep.2019.05.012. 31546158

